# Autophagy-associated circular RNA hsa_circ_0007813 modulates human bladder cancer progression via hsa-miR-361-3p/IGF2R regulation

**DOI:** 10.1038/s41419-021-04053-4

**Published:** 2021-08-07

**Authors:** Zheyu Zhang, Zezhong Mou, Chenyang Xu, Siqi Wu, Xiyu Dai, Xinan Chen, Yuxi Ou, Yiling Chen, Chen Yang, Haowen Jiang

**Affiliations:** 1 Department of Urology, Huashan Hospital, Fudan University, Shanghai, China; 2 Fudan Institute of Urology, Huashan Hospital, Fudan University, Shanghai, China; 3 National Clinical Research Center for Aging and Medicine, Fudan University, Shanghai, China

**Keywords:** Cancer metabolism, Bladder cancer, Macroautophagy, Cell growth, Cell invasion

## Abstract

Circular RNAs (circRNAs) drive several cellular processes including proliferation, survival, and differentiation. Here, we identified a circRNA hsa_circ_0007813, whose expression was upregulated in bladder cancer. High hsa_circ_0007813 expression was associated with larger tumor size, higher primary tumor T stage, and higher pathologic grade. Survival analysis showed that patients with high hsa_circ_0007813 expression levels had a poorer prognosis. Based on these findings from clinical tissue samples and cell lines, we assumed that hsa_circ_0007813 functioned a vital role in bladder cancer progression. Next, functional experiments revealed that knockdown of hsa_circ_0007813 inhibited proliferation, migration, and invasiveness of bladder cancer cells both in vitro and in vivo. Through extensive bioinformatic prediction and RNA pull-down assays, we identified hsa-miR-361-3p as a competing endogenous RNA of hsa_circ_0007813. Further bioinformatic studies narrowed targets to 35 possible downstream genes. We then found that knockdown of hsa_circ_0007813 led to altered cell autophagy, bringing our attention to IGF2R, one of the possible downstream genes. IGF2R was also known as cation-independent mannose-6-phosphate receptor (CI-M6PR), was discovered to participate in both autophagy and tumor biology. Regarding autophagy has a dominant role in the survival of tumor cells overcoming cellular stress and correlates with tumor progression, investigations were made to prove that hsa_circ_0007813 could regulate IGF2R expression via hsa-miR-361-3p sponging. The potential of hsa_circ_0007813 in regulating IGF2R expression explained its influence on cell behavior and clinical outcomes. Collectively, our data could offer new insight into the biology of circRNA in bladder cancer.

## Introduction

Bladder cancer was estimated to be the fourth leading incidence type of cancer in the male population, causing 81400 new cases and 17980 deaths in the United States in 2020 [1]. Although efforts have been made to improve the treatment techniques, the mortality of invasive bladder cancer cases didn’t decrease sharply. A better knowledge of molecular mechanisms contributing to the tumor progression and invasiveness may help improving treatment methods of bladder cancer.

Circular RNAs (circRNAs), first discovered in 1976, have been identified as a novel kind of endogenous noncoding RNAs (ncRNAs) constructed via shearing closed loop structures derived from its formal linear transcript without 5′ caps or 3′ polyadenylated tails [2, 3]. Additionally, it has been revealed that circRNAs drive several cellular processes including proliferation, survival, and differentiation [4]. It has already been reported that circRNAs, such as circ-ITCH [5], circHIPK3 [6], and circCASC15 [7] contribute to human bladder cancer biology.

Autophagy, a highly evolutionarily conserved metabolic process which functions as a response to cellular stresses via degrading eukaryotic cellular components as organelles as well as pathogens [8]. Autophagy is dysregulated in several diseases, including cancer. Autophagy has a dominant role in the survival of tumor cells overcoming cellular stress and correlates with tumor progression and chemotherapy resistance [9]. Several circRNAs have been identified as autophagy-associated mediators in cancer [10, 11], however, the functional implications of autophagy-associated circRNAs in bladder cancer still remain unclear.

Here, we identified an upregulated circRNA hsa_circ_0007813 in bladder cancer. Through extensive bioinformatic prediction and RNA pull-down assays, we identified hsa-miR-361-3p as a competing endogenous RNA of hsa_circ_0007813. Further bioinformatic studies narrowed targets to 35 possible downstream genes. We then found that knockdown of hsa_circ_0007813 led to altered cell autophagy, bringing our attention to IGF2R, one of the possible downstream genes. IGF2R, also known as cation-independent mannose-6-phosphate receptor (CI-M6PR), was discovered to participate in both autophagy and tumor biology. IGF2R is localized mostly in the Golgi and endosomal compartments with less than 10% on the plasma membrane. It was proved that knockdown of IGF2R could suppress tumorigenic properties of the tumor, as well as tumor cell autophagy [12–14]. In this study, we found that knockdown of hsa_circ_0007813 led to decreased IGF2R expression level, thus leading to decreased tumor proliferation, migration, and invasiveness, as well as autophagy.

## Materials and methods

### Total RNA isolation

Total RNA was isolated from tissues or cells using TRIzol Reagent (Thermo Fisher Scientific, Invitrogen) following the manufacturer’s protocol. Optical density (OD) at 230, 260, and 280 nm were measured by NanoDrop ND-1000 (Thermo Fisher Scientific). OD260/OD280 ratios between 1.8 and 2.1 were deemed acceptable, and OD260/OD230 ratios greater than 1.8 were deemed acceptable.

### Patient samples and circRNA sequencing

Bladder cancer and paired normal samples were obtained under approval from the Ethics Committee of Huashan Hospital. Samples were harvested and immediately snap-frozen in liquid nitrogen. The total RNAs from tissues were treated with RibiZero rRNA Removal Kit (Epicentre, USA). Followed by standard procedures, samples were subjected to Hiseq2000 (Illumina, USA). Fragments were mapped to the human reference genome (GRCH38/hg38) by TopHat2. Counts data acquired were then normalized and proceeded to pair-wise differential gene expression analysis. CircRNAs with *P* value < 0.05 and |log2FC| > 2 were deemed differentially expressed.

### Cell culture and infection

Bladder transitional cell carcinoma cell lines, immortalized uroepithelium cell line, and human embryonic kidney cells (HEK-293) were received from Shanghai Yuanye (Shanghai, China). All cells were maintained at 37 °C and 5% CO_2_ in culture with Dulbecco’s modified Eagle medium (DMEM; Gibco, USA) supplemented with 10% fetal bovine serum (FBS; Gibco, USA).

To overexpress IGF2R, the recombinant plasmid vectors pENTER carrying IGF2R ORF or control ORF was purchased from Vigene (China). HEK-293 cells were transfected with psPAX2 and pMD2.G. The medium containing virus particles was collected and added to the T24 and UM-UC-3. After 48 h, the selection was done with 1.0 µg/ml puromycin (Sangon, China).

### Small interfering RNA, miRNA mimic, and miRNA inhibitor transient transfection

Small interfering RNA, miRNA mimic, and miRNA inhibitor were designed and synthesized by GenePhama (China) (Sequences of RNAs were presented in Supplementary Table S3). Small interfering RNAs (siRNAs), miRNA mimic, and miRNA inhibitor were transfected into cells using siRNA-Mate (GenePharma, China) following standard procedure.

### Cell proliferation assay

Cellular proliferation was assessed with Cell Counting Kit-8 (CCK8; Sigma-Aldrich, USA). Cells were seeded into 96-well plates with three replications. OD at 480 nm length was measured after seeding.

For colony formation assay, cells were seeded into six-well plates. After 7–9 days of incubation, cells were fixed with 4% (w/v) paraformaldehyde (PFA) and stained with crystal violet solution.

### Transwell assay

Cell invasiveness potential was analyzed using Transwell chambers (Corning, USA) in accordance with the manufacturer’s protocol. After incubation for 24 h, the cells on the upper surfaces of the Transwell chambers were removed and the cells located on the lower surfaces were fixed with 4% PFA. After staining with crystal violet, cells were photographed and counted in five randomly selected fields.

### Quantitative reverse transcriptase PCR

Quantitative reverse transcriptase PCR (qRT-PCR) of circRNAs and mRNAs was performed using SYBR Premix Ex Taq (TaKaRa, Beijing) following the manufacturer’s protocol. Relative expression of GAPDH was chosen as the reference gene. qRT-PCR of miRNAs was performed using Mir-X miRNA qRT-PCR TB Green Kits (TaKaRa, Beijing) following the manufacturer’s protocol. Relative expression of U6 was chosen as reference miRNA. RNA reverse transcription and quantitative PCR was done with CFX96 Touch real-time (BIORAD, America). Primers were designed and synthesized by GenePhama (China) (Sequences of primers were presented in Supplementary Table S3).

### Western blot analysis

Proteins were separated by 8% SDS-PAGE and transferred to nitrocellulose transfer membranes. The blots were blocked with freshly prepared 5% nonfat milk in TBST for 1 h at room temperature. Then the blots were incubated at 4 °C overnight with primary antibodies. After washing with TBST, the blots were incubated with horseradish peroxidase-conjugated (HRP-conjugated) donkey anti-rabbit IgG or sheep anti-mouse IgG (Invitrogen, China) at room temperature for 1 h. ECL substrate (CLiNX, Shanghai, China) and ChemiScope Touch (CLiNX, Shanghai, China) were used for detecting HRP-conjugated antibodies. Primary antibodies including anti-P-AMPK (phospho S496), anti-P-S6K (phospho S424), and anti-P-AKT (Ser473) were brought from Abcam (China). Primary antibodies including anti-LC3, anti-P-ERK(Thr202/Tyr204), anti-PARP1, anti-vimentin, anti-SQSTM1/p62, anti-GAPDH, and anti-IGF2R(M6PR) were brought from Proteintech (China).

### Immunocytochemistry assays

Cells were seeded on glass coverslips pretreated with TC (Solarbio Life Science, China) and cultured for 24 h. Cells were fixed with 4% PFA at room temperature for 10 min. Then treated with 0.5% Triton X-100 for 5 min. Cells were blocked with 2% BSA/PBST for 1 h and incubated with primary antibodies at 4 °C overnight. The fluorophore-labeled secondary antibody solution was added onto the glass coverslips and incubated for 1 h. Glass coverslips were finally stained and mounted with Antifade Mounting Medium with DAPI (Beyotime, China). Primary antibodies including anti-LC3 and anti-LAMP1 were brought from Proteintech (China).

### In situ hybridization

Tissue sections were digested with 15 μg/ml proteinase K (Yeasen, China) for 20 min at room temperature and then incubated with digoxigenin-labeled RNA probe for 2 h at 55 °C. Three percent H_2_O_2_ was used to inactivate endogenous peroxidases. After incubation with anti-digoxigenin antibody and HRP-conjugated secondary antibody, a tyramine-conjugated fluorochrome (TSA) reaction was performed for 12 min. The tissue slides were then loaded onto ChemiScope 6200 Touch (CLiNX, Shanghai, China) for in situ hybridization analysis. Fluorescent hsa_circ_0007813 probes were designed and synthesized by Genscript (China) (Sequences of probes were presented in Supplementary Table S3).

### Immunohistochemistry

For immunohistochemistry (IHC) staining, formalin-fixed paraffin-embedded (FFPE) tissues were cut into 4-µm-thick slides. Antigen retrieval was achieved by heating the slides in 0.01 M citrate buffer (pH 6.0) for 20 min. Endogenous peroxidase activity was blocked using 3% H_2_O_2_. Primary antibody incubation was next done overnight at 4 °C. The slides were washed with PBS and incubated with secondary antibody for 1 h at room temperature. The slides were washed with PBS and followed by development with 3,3′-diaminobenzidine. The nuclei were stained with Gill’s hematoxylin solution and the slides were then mounted.

### Transplantable xenograft mouse model

Nude mice were maintained under a specific pathogen-free condition with the approval of the Animal Care Committee of Fudan University. FDA regulations of animal research were followed. BALB/c Nude mice (6 weeks old, male) were obtained from SLACOM (Shanghai, China) and used as xenograft hosts. No randomization or blinding method was used.

For the subcutaneous tumor model, 1 × 10^7^ transfected T24 cells were suspended in 0.2 ml of PBS and subcutaneously injected. The mice were subjected to in vivo imaging and sacrificed for dissection 21 days after injection. For metastasis analyses, 1 × 10^4^ transfected cells were intravenously injected into mice tails. After 28 days, mice were subjected to in vivo imaging and sacrificed for dissection.

### In vivo imaging

Mice were intraperitoneally injected 200 mg d-luciferin/kg body weight (Beyotime, Shanghai, China). Then mice were anaesthetized with isoflurane. Bioluminescence imaging was done with the IVIS Spectrum In Vivo Imaging System and Living Image software (PerkinElmer, Waltham, MA).

### RNA-RNA pulldown

Biotin-coupled hsa_circ_0007813 probes were designed and synthesized by Genscript (China) (Sequences of probes were presented in Supplementary Table S3). CircRNA-miRNA pulldown was performed using streptavidin-coupled magnetic beads (Life Technologies, USA), which were incubated with biotin-coupled hsa_circ_0007813 probes or oligo probes for 3 h at room temperature. Cell lysates were incubated with probe-coated beads at 4 °C overnight.

### Luciferase reporter assay

Wild-type (WT) cDNA fragments with predicted hsa-miR-361-3p binding site of hsa_circ_0007813 and IGF2R 3′-untranslated region (3′-UTR) were amplified by PCR. Mutated fragments (MUT) of these fragments were acquired by overlap extension PCR. Next, WT and MUT fragments were recombined into psiCHECK-2 (Promega, Madison, WI, USA). Luciferase activity was measured through a dual-luciferase reporter assay kit (Promega, China).

### Bioinformatics analysis

RNA interaction prediction tools including miRanda [15], Circular RNA Interactome [16], and CircBank Database [17] were enrolled to help finding miRNA–circRNA interactions. For miRNA–gene interactions, we retrieved information from miRWalk2.0 [18]. We selected the cases predicted by eight algorithms which included miRWalk, Microt4, miRMap, Pictar2, PITA, RNA22, RNAhybrid, and Targetscan. We used Cytoscape to export the diagram.

Gene expression grouped survival analysis for samples from the cancer genome atlas (TCGA) was carried out and visualized by Gene expression profiling interactive analysis (GEPIA) [19].

### Statistical analyses

Quantitative data of western blot analysis was acquired by ImageJ (Fiji). Statistical analyses were performed only when a minimum of three independent samples were acquired and the variance was assessed. Statistical analysis was performed with IBM SPSS Statistics 26 software. Log-rank test, Chi-Squared test, Kruskal–Wallis test, Wilcoxon matched-pairs signed-rank test, generalized least squares, and *F*-test were performed, as indicated in figure legends. Results were considered statistically significant when *P < 0.05, **P < 0.01, ***P < 0.001. All error bars in the figures indicated the standard deviation of three independent experiments.

## Results

### High hsa_circ_0007813 expression predicted unfavorable prognosis in bladder cancer patients

**Fig. 1:**
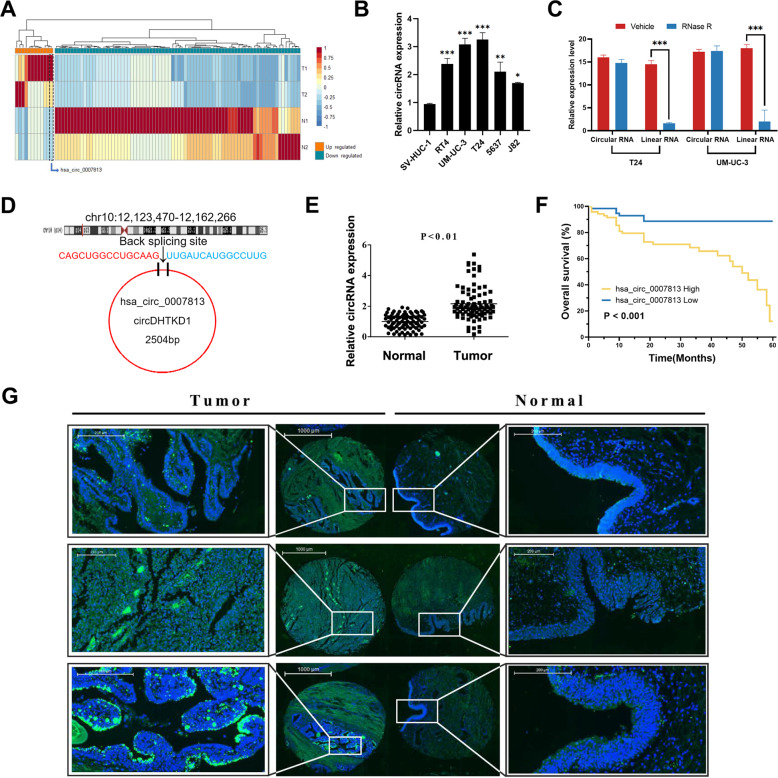
High hsa_circ_0007813 expression predicted unfavorable prognosis in bladder cancer patients. **A** Heatmap illustrated expression patterns of differentially expressed circRNAs between two bladder cancer tissues (T1 and T2) and their paired adjacent normal tissues (N1 and N2). **B** Relative hsa_circ_0007813 expression was upregulated in five bladder transitional cell carcinoma cell lines (RT4, UM-UC-3, T24, 5637, and J82), comparing with immortalized uroepithelium cell line SV-HUC-1 (*N* = 3, Kruskal–Wallis test). **C** Total cell RNA from T24 and UM-UC-3 was treated with or without RNase R. Expression level of hsa_circ_0007813 and its linear form was measured by qRT-PCR (*N* = 3, Kruskal–Wallis test). **D** Diagram of the back-splicing site of hsa_circ_0007813. **E** FISH for hsa_circ_0007813 in TMA showed that expression of hsa_circ_0007813 was higher in tumor tissues (*N* = 90, Wilcoxon matched-pairs signed-rank test). **F** Kaplan–Meier survival plot for bladder cancer patients’ overall survival, grouped by median hsa_circ_0007813 expression level (*N* = 90, Logrank test). **G** Representative areas of FISH in TMA were selected to show hsa_circ_0007813 expression between paired tumor and normal samples (Green: hsa_circ_0007813, Blue: DAPI, scale bars represent 1000 μm). All error bars in the figures indicated the standard deviation of three independent experiments. *P < 0.05, **P < 0.01, ***P < 0.001.

In an attempt to screen circRNAs with biological significance, we performed circRNA high-throughput sequencing on total RNA from two human bladder cancer tissues and paired normal bladder epithelium tissues. Patient No. 1 was a 51-year-old male, with pathologically diagnosed high grade (2004 WHO/ISUP) muscle-invasive bladder cancer, staged T2aN0M0 (AJCC eighth edition). Patient No. 2 was a 56-year-old male, with pathologically diagnosed high-grade muscle-invasive bladder cancer, staged T2bN0M0. A total of 12 circRNAs were significantly upregulated and 78 circRNAs were significantly downregulated (Filtered by |Log2FC| ≥ 2 and adjusted-*P* < 0.05). All the differentially expressed circRNAs were illustrated by heatmap with hierarchical clustering analysis (Fig. 1A). As our result was carried out from two pairs of samples, a random error could cause false-positive findings. We then pick the upregulated circRNAs with the top 50% Log2FC, and ranked them by Log2FC divided by the deviation of CPM values of two tumor samples (Supplementary Table S1). Among them, hsa_circ_0007813 got a high value and was not studied in bladder cancer yet.

According to the sequencing result, hsa_circ_0007813 was upregulated in bladder cancer tissues (Log2FC = 6.10, *P* = 0.037). Next, qRT-PCR was carried out across uroepithelium cell lines to further validate the previous sequencing obtained findings. Higher relative hsa_circ_0007813 expression was observed in five bladder transitional cell carcinoma cell lines comparing to immortalized uroepithelium cell line (Fig. 1B). Due to circular RNA’s circulated loop structure, it gains the ability to resist Ribonuclease R (RNase R) treatment. We found that RNase R decreased hsa_circ_0007813 linear RNA form levels in UM-UC-3 and T24, but it did not affect circular RNA form levels (Fig. 1C). CircRNA hsa_circ_0007813 is derived from circularized exons from gene DHTKD1, which is located at chr10:12123470–12162266. The length of spliced mature circRNA is 2504 bp (Fig. 1D). We next examined the associations of hsa_circ_0007813 expression level with clinical and pathologic features by using tissue microarray (TMA), which included 90 bladder cancer tissues and its paired adjacent normal tissues. Fluorescent in situ hybridization (FISH) assays demonstrated that hsa_circ_0007813 expression was higher in bladder cancer tissues (Fig. 1E). Representative results of FISH in TMA were selected to show different hsa_circ_0007813 expression patterns in paired tumor and normal samples (Fig. 1G). Associations of hsa_circ_0007813 expression level with clinical and pathologic features were summarized in Table 1. High hsa_circ_0007813 expression was associated with larger tumor size (≥3 cm), higher primary tumor T stage (pT2, pT3, and pT4), and higher pathologic grade (high grade). Survival analysis between hsa_circ_0007813 high expression patients and hsa_circ_0007813 low expression patients was carried out. Results showed that patients with high hsa_circ_0007813 expression levels had a poorer prognosis (Fig. 1F). Based on these findings from clinical tissue samples and cell lines, we assumed that hsa_circ_0007813 functioned a vital role in bladder cancer progression.

**Table 1:** Associations of hsa-circ-0007813 expression level with clinical and pathologic features for 90 bladder cancer patients.

	All patients	hsa-circ-0007813 expression level		
		Low	High	
	*N* = 90	*N* = 28	*N* = 62	
Characteristic	*N* (%)			*P* value
**Gender**				
Male	81 (90.0)	22 (78.5)	59 (95.1)	0.024
Female	9 (10.0)	6 (21.4)	3 (4.8)	
**Age at surgery (years)**				
<65	56 (62.2)	18 (64.2)	38 (61.2)	0.819
≥65	34 (37.7)	10 (35.7)	24 (38.7)	
**Tumor size**				
<3 cm	38 (42.2)	20 (71.4)	18 (29.0)	<0.001
≥3 cm	52 (57.7)	8 (28.5)	44 (70.9)	
**Primary tumor T stage (TNM 8th ed)**				
pTa, Tis and pT1	61 (67.7)	24 (85.7)	37 (59.6)	0.016
pT2, pT3 and pT4	29 (32.2)	4 (14.2)	25 (40.3)	
**Regional lymph node involvement (TNM 8th ed)**				
pN1, pN2, and pN3	22 (24.4)	10 (35.7)	12 (19.3)	0.115
pN0	68 (75.5)	18 (64.2)	50 (80.6)	
**Grade (WHO/ISUP 2004)**				
Low grade	51 (56.6)	23 (82.1)	28 (45.1)	0.001
High grade	39 (43.3)	5 (17.8)	34 (54.8)	

### Silencing hsa_circ_0007813 suppressed proliferation, migration, and invasiveness potentials of bladder cancer cells in vitro and in vivo

**Fig. 2:**
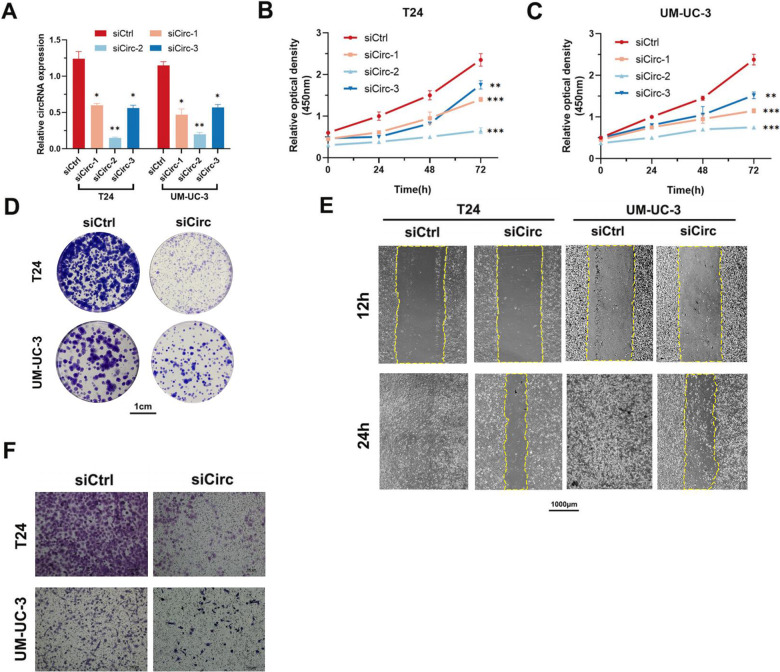
Silencing hsa_circ_0007813 suppressed proliferation, migration, and invasiveness potentials of bladder cancer cells in vitro. **A** At 48 h after siRNA (si-circ-1, si-circ-2, and si-circ-3) transfection, hsa_circ_0007813 expression levels in both T24 and UM-UC-3 were analyzed by qRT-PCR (*N* = 3, Kruskal–Wallis test). At 24 h after siRNA transfection, **B**, **C** CCK-8 assays (*N* = 3, Kruskal–Wallis test) and **D** colony formation assays (scale bar represents 1 cm) were used to assess the proliferation ability of cells. **E** Wound healing assays (scale bar represents 1000 μm) were used to assess the migration ability of cells. **F** Transwell assays (scale bar represents 100 μm) were used to assess the invasiveness of cells. All error bars in the figures indicated the standard deviation of three independent experiments. *P < 0.05, **P < 0.01, ***P < 0.001.

Three small interference RNAs (siRNAs) targeting hsa_circ_0007813 were designed. While all siRNA reduced hsa_circ_0007813 expression in bladder cancer cells, one of them showed the highest knockdown effect (Fig. 2A). Furthermore, Cell Counting Kit-8 (CCK-8) assays showed that knockdown of hsa_circ_0007813 via siCirc-2 significantly inhibited proliferation of T24 and UM-UC-3 (Fig. 2B, C). Colony formation assays also showed that silencing hsa_circ_0007813 suppressed cell proliferation of UM-UC-3 and T24 cells (Fig. 2D). Wound healing assays (Fig. 2E) and Transwell assays (Fig. 2F) showed that silencing hsa_circ_0007813 also suppressed cell migration and invasiveness of UM-UC-3 and T24 cells.

**Fig. 3:**
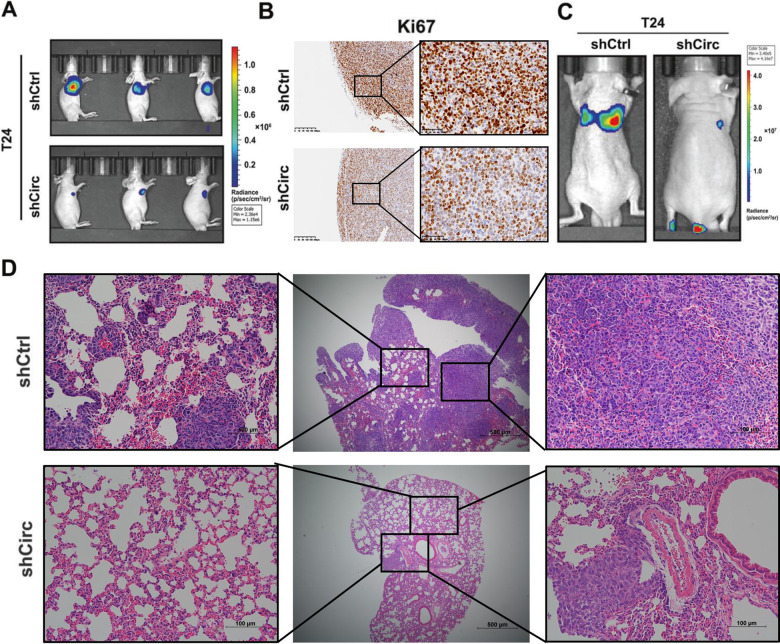
Silencing hsa_circ_0007813 suppressed proliferation, migration, and invasiveness potentials of bladder cancer cells in vivo. **A** Luciferase expressing T24 transfected with control shRNA (shCtrl) and shRNA (shCirc) were used to establish subcutaneous xenograft models. In vivo bioluminescence imaging was used to monitor tumor growth (*P* < 0.05, *N* = 3, Kruskal–Wallis test). **B** Ki67 IHC staining of tumor tissues dissected from subcutaneous xenograft models (Scale bars represent 100 μm). **C** Bioluminescence imaging of mice metastasis models (*N* = 1). **D** Representative H&E-stained slices of lungs taken from shCtrl and shCirc bearing mice (Scale bars in the second column represent 500 μm; Scale bars in the first and third columns represent 100 μm). All error bars in the figures indicated the standard deviation of three independent experiments. *P < 0.05, **P < 0.01, ***P < 0.001.

We then investigated the effects of hsa_circ_0007813 knockdown on tumor growth and metastasis in vivo. Luciferase expressing T24 cell subcutaneous xenograft models showed that hsa_circ_0007813 knockdown suppressed bladder cancer tumor growth in vivo (Fig. 3A). Immunohistochemical (IHC) staining of Ki67 verified the effect of hsa_circ_0007813 on tumor growth (Fig. 3B). Next, control and hsa_circ_0007813 knockdown T24 cells were injected into the tail vein of nude mice to assess the metastasis ability of hsa_circ_0007813. Bioluminescence imaging revealed that silencing hsa_circ_0007813 reduced the metastasis potential of T24 cells (Fig. 3C). In consist with the bioluminescence imaging results, hematoxylin and eosin-stained (H&E-stained) slices showed that circRNA knockdown T24 cells formed up fewer and smaller lesion nodules in lungs (Fig. 3D). Taken together, these results suggested that silencing hsa_circ_0007813 suppressed proliferation, migration, and invasiveness potentials of bladder cancer cells in vitro and in vivo.

### CircRNA hsa_circ_0007813 acted as a sponge for hsa-miR-361-3p, a micro-RNA targeting IGF2R

**Fig. 4:**
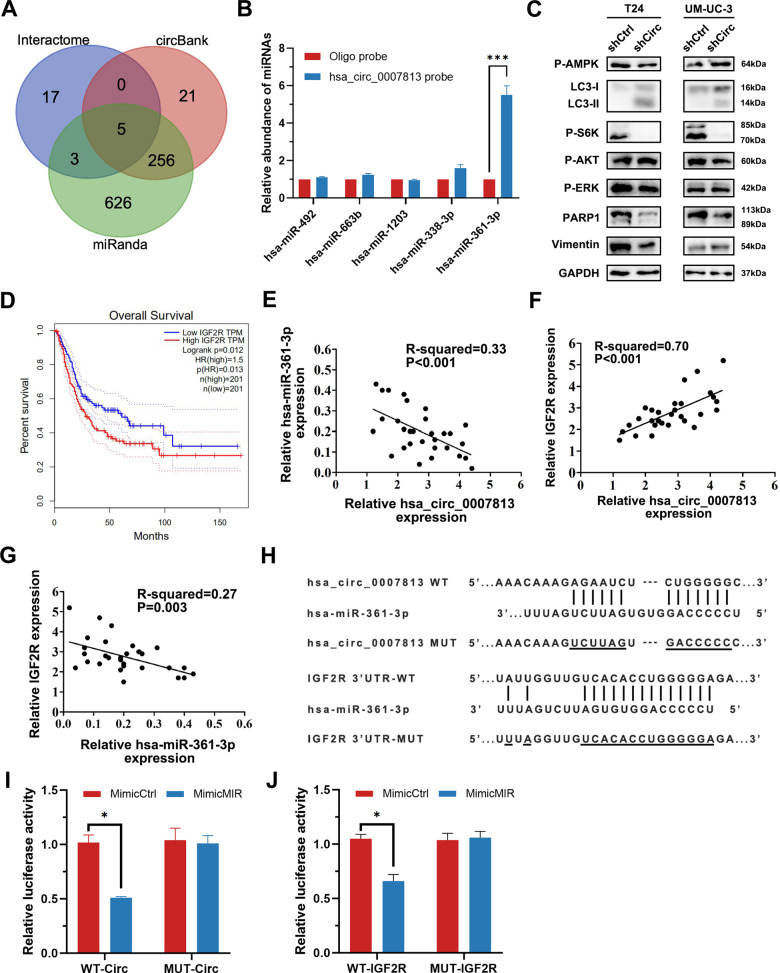
CircRNA hsa_circ_0007813 acted as a sponge for hsa-miR-361-3p, a micro-RNA targeting IGF2R. **A** Venn diagram showed potential downstream miRNA targets of hsa_circ_0007813. The prediction was carried out by interactome, circBank, and miRanda. **B** RNA-RNA pulldown assays were carried out to determine the downstream miRNA (*N* = 3, Kruskal–Wallis test). **C** Control and hsa_circ_0007813 knockdown cells were subjected to western blot analysis with anti-P-AMPK, anti-LC3, anti-P-S6K, anti-P-AKT, anti-P-ERK, anti-PARP1, anti-Vimentin, and anti-GAPDH. GAPDH was used as loading controls. **D** Kaplan–Meier plot of bladder cancer patients’ overall survival, grouped by median IGF2R expression level (Carried out by GEPIA). **E** Scatter plot presenting correlated expression levels between hsa_circ_0007813 and hsa-miR-361-3p in bladder cancer tissues (*N* = 30, generalized least squares and *F*-test). **F** Scatter plot presenting correlated expression levels between hsa_circ_0007813 and IGF2R in bladder cancer tissues (*N* = 30, generalized least squares and *F*-test). **G** Scatter plot presenting correlated expression levels between hsa-miR-361-3p and IGF2R (*N* = 30, generalized least squares and *F*-test). **H** Potential binding sites of hsa_circ_0007813/hsa-miR-361-3p and IGF2R mRNA 3′-UTR/hsa-miR-361-3p were located by Interactome. The third and sixth rows showed mutated hsa_circ_0007813 (MUT) and IGF2R mRNA 3′-UTR (MUT) sequence for dual-luciferase reporter assay. **I** HEK-293 cells were transfected with dual-luciferase reporter vectors carrying wild-type (WT-Circ) or mutated (MUT-Circ) hsa_circ_0007813 fragments. Cells were then transfected with control miRNA mimic (MimicCtrl) or hsa-miR-361-3p mimic (MimicMIR). At 48 h after transfection, luciferase activities were measured (*N* = 3, Kruskal–Wallis test). **J** HEK-293 cells were transfected with dual-luciferase reporter vectors carrying wildtype (WT-IGF2R) or mutated (MUT-IGF2R) IGF2R mRNA 3’-UTR fragments. Cells were then transfected with control miRNA mimic (MimicCtrl) or hsa-miR-361-3p mimic (MimicMIR). At 48 h after transfection, luciferase activities were measured (*N* = 3, Kruskal–Wallis test). All error bars in the figures indicated the standard deviation of three independent experiments. *P < 0.05, **P < 0.01, ***P < 0.001.

CircRNAs regulate the cell biology process mainly by influencing miRNA-induced mRNA degradation. RNA interaction prediction tools including Circular RNA Interactome, miRanda, and circBank Database were used to screen potential targets of hsa_circ_0007813. Five miRNAs (hsa-miR-492, hsa-miR-663b, hsa-miR-1203, hsa-miR-338-3p, and hsa-miR-361-3p) were overlapped in all the prediction methods we chose (Fig. 4A). By using circRNA-miRNA pull-down assay, we found that hsa-miR-361-3p was able to interact with hsa_circ_0007813 (Fig. 4B). Next, we screened downstream genes of hsa-miR-361-3p by using eight miRNA–gene interaction prediction software (miRWalk, Microt4, miRMap, Pictar2, PITA, RNA22, RNAhybrid, and Targetscan). After filtering by eight software, we focused on 35 genes. Next, we conducted TCGA-BLCA samples survival analysis throughout the 35 genes, and ranked them by Benjamini & Hochberg adjusted *P* value. The top five genes after ranking were EMP1, IGF2R, FAIM2, SOX2, and BTG2 (Supplementary Table S2). To narrow our search further, multiple cell signaling pathways indicator including Phospho-AMPK (P-AMPK), Phospho-S6K (P-S6K), Phospho-AKT (P-AKT), Phospho-ERK (P-ERK), PARP1, Vimentin, and LC3 were analyzed in control and hsa_circ_0007813 knockdown cells (Fig. 4C). An increased LC3-II level was observed in hsa_circ_0007813 knockdown cells, indicating the upregulated formation of the autophagosome. Thinking of the top five genes after ranking, we noticed IGF2R, a member of the IGF family. Recent work in cervical cancer suggested that IGF2R knockdown had an autophagic inhibitory effect [13]. Thus, we supposed the regulative effect of hsa_circ_0007813 on autophagy was mediated by IGF2R. Previous survival analysis of TCGA-BLCA samples was shown in the figure, indicating that high IGF2R expression level predicted poor bladder cancer OS (Fig. 4D).

Using qRT-PCR, we next figured out that relative hsa_circ_0007813 expression level was negatively correlated with hsa-miR-361-3p level (Fig. 4E), and positively correlated with IGF2R level (Fig. 4F), in bladder cancer tissues. Likewise, the relative hsa-miR-361-3p expression level was negatively correlated with the IGF2R level (Fig. 4G). We then conducted luciferase assays. The predicted binding sites and mutated sequences of hsa_circ_0007813 and hsa-miR-361-3p, as well as hsa-miR-361-3p and IGF2R were presented in figures (Fig. 4H). Luciferase activity of HEK-293 cells carrying wild-type hsa_circ_0007813 reporter vectors could be lowered by treating cells with hsa-miR-361-3p mimic. However, luciferase activity was not lowered in cells carrying mutated hsa_circ_0007813 reporter vectors (Fig. 4I). Similar findings were obtained when conducted with WT-IGF2R and MUT-IGF2R reporter vectors (Fig. 4J). The above results demonstrated that hsa-miR-361-3p could both bind to hsa_circ_0007813 and IGF2R mRNA 3′-UTR.

To investigate the relationship among hsa_circ_0007813, hsa-miR-361-3p, and IGF2R, we transfected T24 and UM-UC-3 cells with hsa-miR-361-3p miRNA inhibitor after hsa_circ_0007813 knockdown. These cells were next subjected to CCK-8 assays (Supplementary Fig. S1A, D), colony formation assays (Supplementary Fig. S1G), wound healing assays (Supplementary Fig. S1J), and Transwell assays (Supplementary Fig. S1M). Results showed that the proliferation, migration, and invasiveness inhibitory effects caused by hsa_circ_0007813 knockdown could be rescued by hsa-miR-361-3p knockdown. The siRNA-transfected cells were further transfected with IGF2R overexpression vectors. Results showed that the proliferation, migration, and invasiveness inhibitory effects caused by hsa_circ_0007813 knockdown could likewise be rescued by IGF2R overexpression Supplementary Fig. S1B, E, H, K, N). Furthermore, after hsa-miR-361-3p miRNA mimic transfection, cells were further transfected with IGF2R overexpression vectors. Results revealed that the proliferation, migration, and invasiveness inhibitory effects caused by hsa-miR-361-3p overexpression could be rescued by IGF2R overexpression (Supplementary Fig. S1C, F, I, L, O). To sum it up, circRNA hsa_circ_0007813 acted as a sponge for hsa-miR-361-3p, a micro-RNA targeting IGF2R.

### CircRNA hsa_circ_0007813 regulated autophagy through hsa-miR-361-3p/IGF2R

**Fig. 5:**
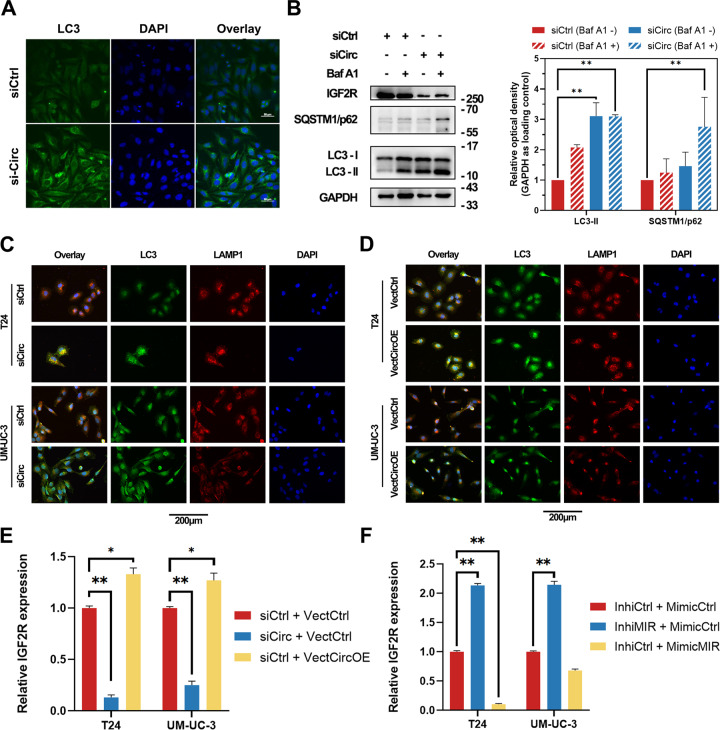
CircRNA hsa_circ_0007813 regulated autophagy through hsa-miR-361-3p/IGF2R. **A** Immunocytochemical analysis of autophagosomes in UM-UC-3 cells transfected with siRNA (siCirc) or control siRNA (siCtrl). **B** At 48 h after siRNA transfection, UM-UC-3 cells were treated with Bafilomycin A1 (Baf A1) 50 nM or DMSO for 24 h. Western blot analysis showed the expression level of SQSTM/p62, LC3-I, and LC3-II. GAPDH was used as loading controls (Left panel). Quantitative analysis was conducted (Right panel, *N* = 3, Kruskal–Wallis test). **C** Immunocytochemical analysis of autophagosomes and lysosomes in T24 and UM-UC-3 cells transfected with siRNA (siCirc) or control siRNA (siCtrl). Anti-LC3 and anti-LAMP1 antibodies were used to mark autophagosomes (Green) and lysosomes (Red), respectively. **D** Immunocytochemical analysis of autophagosomes and lysosomes in T24 and UM-UC-3 cells transfected with hsa_circ_0007813 overexpression vectors (VectCircOE) or control vectors (VectCtrl). Anti-LC3 and anti-LAMP1 antibodies were used to mark autophagosomes (Green) and lysosomes (Red), respectively. The RNA expression profiles of IGF2R were measured by qRT-PCR in (**E**) hsa_circ_0007813 knockdown and overexpression cells (*N* = 3, Kruskal–Wallis test), as well as (**F**) hsa-miR-361-3p downregulated and upregulated cells (*N* = 3, Kruskal–Wallis test). All error bars in the figures indicated the standard deviation of three independent experiments. *P < 0.05, **P < 0.01, ***P < 0.001.

According to the previous findings, circRNA hsa_circ_0007813 could positively regulate IGF2R, a gene found to has an autophagy-regulatory function. Immunocytochemical analysis of autophagosomes in UM-UC-3 cells revealed that knockdown of hsa_circ_0007813 also had an autophagy-regulatory function in bladder cancer cells (Fig. 5A). For autophagic flux analysis, we treated cells with Bafilomycin A1, an inhibitor of lysosome acidification. As Bafilomycin A1 blocks late autophagy, in the condition of Bafilomycin A1 treatment, the LC3-II expression level indicates the autophagosomes in cells. Western blot results revealed that Bafilomycin A1 induced LC3-II upregulation was not enhanced by hsa_circ_0007813 knockdown, suggesting that this knockdown had an autophagic inhibitory effect. We also observed a slight but not significant rise of SQSTM1/p62 expression in circRNA knockdown cells (Fig. 5B). Using immunocytochemical analysis, we noticed promoted autophagosome formation in both hsa_circ_0007813 knockdown (Fig. 5C) and overexpression cells (Fig. 5D), suggesting the autophagy-regulatory function of hsa_circ_0007813. RNA expression profiles of IGF2R in hsa_circ_0007813/hsa-miR-361-3p knockdown and overexpression cells were carried out by qRT-PCR (Fig. 5E, F). Higher IGF2R expression levels were observed in either hsa_circ_0007813 overexpression cells or hsa-miR-361-3p knockdown cells, while lower IGF2R expression levels were observed in either hsa_circ_0007813 knockdown cells or hsa-miR-361-3p overexpression cells.

**Fig. 6:**
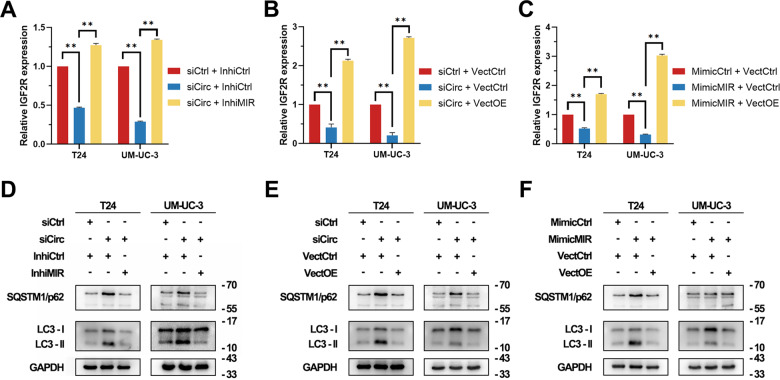
CircRNA hsa_circ_0007813 regulated autophagy through hsa-miR-361-3p/IGF2R. The siRNA-transfected cells (siCirc) were further transfected with hsa-miR-361-3p miRNA inhibitor (InhiMIR). These cells were subjected to **A** qRT-PCR (N = 3, Kruskal–Wallis test) and **D** western blot analysis assays. The siRNA-transfected cells (siCirc) were further transfected with IGF2R overexpression vectors (VectOE). These cells were subjected to **B** qRT-PCR (*N* = 3, Kruskal–Wallis test) and **E** western blot analysis assays. After hsa-miR-361-3p miRNA mimic (MimicMIR) transfection, cells were further transfected with IGF2R overexpression vectors (VectOE). These cells were subjected to **C** qRT-PCR (*N* = 3, Kruskal–Wallis test) and **F** western blot analysis assays. GAPDH was used as a loading control. All error bars in the figures indicated the standard deviation of three independent experiments. *P < 0.05, **P < 0.01, ***P < 0.001.

We next designed rescue experiments in T24 and UM-UC-3 cells. By using qRT-PCR, expression levels of IGF2R were measured (Fig. 6A, B, C). Meanwhile, expression levels of LC3-II and SQSTM1/p62 were assessed by western blot. It was shown that the inhibitory effects of hsa_circ_0007813 knockdown on IGF2R expression and autophagy could be rescued by hsa-miR-361-3p knockdown or IGF2R overexpression (Fig. 6D, E). The inhibitory effects of hsa-miR-361-3p overexpression on IGF2R expression and autophagy could be rescued by IGF2R overexpression (Fig. 6F). Overall, we demonstrated that knockdown of hsa_circ_0007813 has an inhibitory effect on IGF2R expression and autophagy.

## Discussion

CircRNA sequencing, clinical data, experimental models, and bioinformatics prediction, we elucidated aspects of the underlying biology of hsa_circ_0007813 that could regulate bladder cancer biological behavior. Our sequencing results showed that hsa_circ_0007813 was upregulated in bladder cancer tissues. Using qRT-PCR and FISH, we next confirmed that hsa_circ_0007813 was overexpressed in bladder cancer cells and its high expression level was associated with a poor survival rate. Through siRNA knockdown, we found that silencing hsa_circ_0007813 suppressed proliferation, migration, and invasiveness potential of bladder cancer cells in vitro, as well as in vivo. It was only in hepatocellular carcinoma that hsa_circ_0007813 was described to be upregulated in tumor tissues and may be associated with tumor progression [20], let alone its biology in bladder cancer. CircRNAs play important roles in regulating mRNAs at the transcriptional or posttranscriptional level: binding to miRNAs by a competing endogenous RNAs mechanism to block the inhibition of the target gene expression [4]. Our dual-luciferase and functional recovery studies next revealed that hsa_circ_0007813 could act as a sponge for hsa-miR-361-3p, thus regulate IGF2R. Another study suggested that hsa-miR-361-3p could be sponged by circRNAs and play a tumor inhibitory role in colorectal cancer [21], which made an agreement with our findings. Additionally, through immunocytochemical and western blot analysis, we found that the knockdown of hsa_circ_0007813 has an autophagy inhibitory effect.

Autophagy is an evolutionarily conserved process that involves the degradation of damaged or redundant proteins and dysfunctional cellular components [22]. It was known that autophagy could promote the growth of advanced cancer including lung, breast, pancreas, colorectal, and prostate cancer [23]. Studies reported that Atg7 suppressed p53 activation in K-ras-induced lung cancer, which contributes to tumor growth and progression [24]. Likewise, deficiency of core autophagy genes like Atg7 [25], Atg5 [26], and Fip200 [27] represented tumor suppression effects. Despite suppressing p53, the underlying biology of this includes preventing energy crisis [28], cell death, and antitumor immune response [24, 27, 29]. Moreover, autophagy can be an effective cancer escape mechanism and has been implicated in the development of resistance in bladder cancer [30]. Activation of autophagy was reported to help bladder cancer cells resisting cisplatin treatment [31], which could participate in clinical outcomes.

IGF2R, also known as cation-independent mannose-6-phosphate receptor (CI-M6PR), was discovered to participate in both autophagy and tumor biology. The IGF2R endocytosis is the main pathway for delivery of rh-α-Gal A to the lysosome, a kind of lysosome-function-needed enzyme [32]. Loss of IGF2R induces lysosome dysfunction and inhibits autophagy [13, 33]. Abnormalities in IGF-II/IGF2R signaling are related to tumor development [34]. Knockdown of IGF2R was found to suppress tumorigenic properties of tumors [12–14]. As our results proved the regulatory axis of hsa_circ_0007813/hsa-miR-361-3p/IGF2R, it was adequate to explain the tumor-promoting and autophagy-promoting effects of hsa_circ_0007813.

In conclusion, our study showed that hsa_circ_0007813 was upregulated in bladder cancer, and it can efficiently sponge hsa-miR-361-3p to regulate IGF2R expression. We also demonstrated that hsa_circ_0007813 knockdown could hinder autophagy and IGF2R expression, which underlay the tumor inhibitory effect of it. Our findings provide a new circRNA biology in bladder cancer.

## Supplementary information

Supplementary tables

Supplementary figure S1

Legend of supplementary figure S1

## Data Availability

The datasets used and/or analyzed during the current study are available from the corresponding author on reasonable request.
