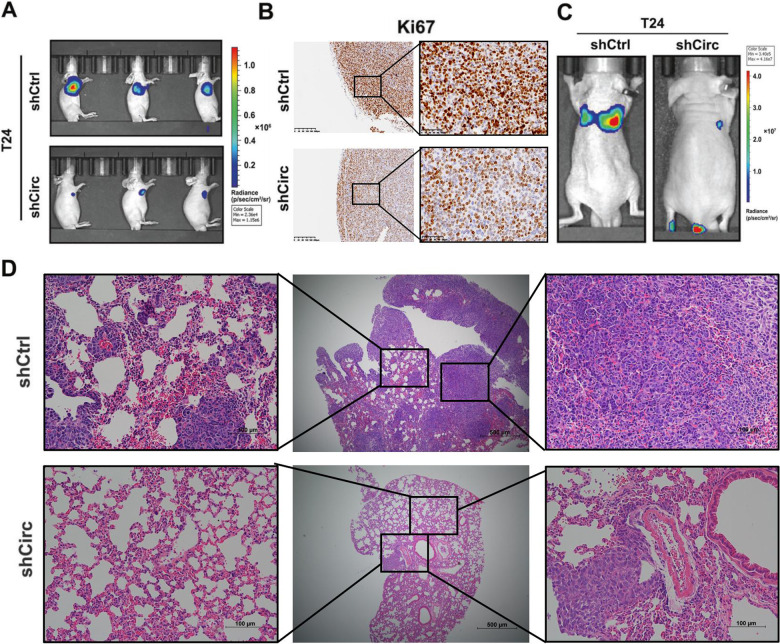# Correction: Autophagy-associated circular RNA hsa_circ_0007813 modulates human bladder cancer progression via hsa-miR-361-3p/IGF2R regulation

**DOI:** 10.1038/s41419-023-05659-6

**Published:** 2023-02-13

**Authors:** Zheyu Zhang, Zezhong Mou, Chenyang Xu, Siqi Wu, Xiyu Dai, Xinan Chen, Yuxi Ou, Yiling Chen, Chen Yang, Haowen Jiang

**Affiliations:** 1 Department of Urology, Huashan Hospital, Fudan University, Shanghai, China; 2 Fudan Institute of Urology, Huashan Hospital, Fudan University, Shanghai, China; 3 National Clinical Research Center for Aging and Medicine, Fudan University, Shanghai, China

**Keywords:** Cancer metabolism, Bladder cancer, Macroautophagy, Cell growth, Cell invasion

Correction to: *Cell Death and Disease* 10.1038/s41419-021-04053-4, published online 07 August 2021

The original version of this article contained an error. In Figs. 2E, F & 3B, "UMUC-3 sicirc" group of Transwell and wound healing assay and the Ki67 staining were wrong. The authors corrected these mistakes. The correct figure can be found below. The original article has been corrected.

**Figure:**
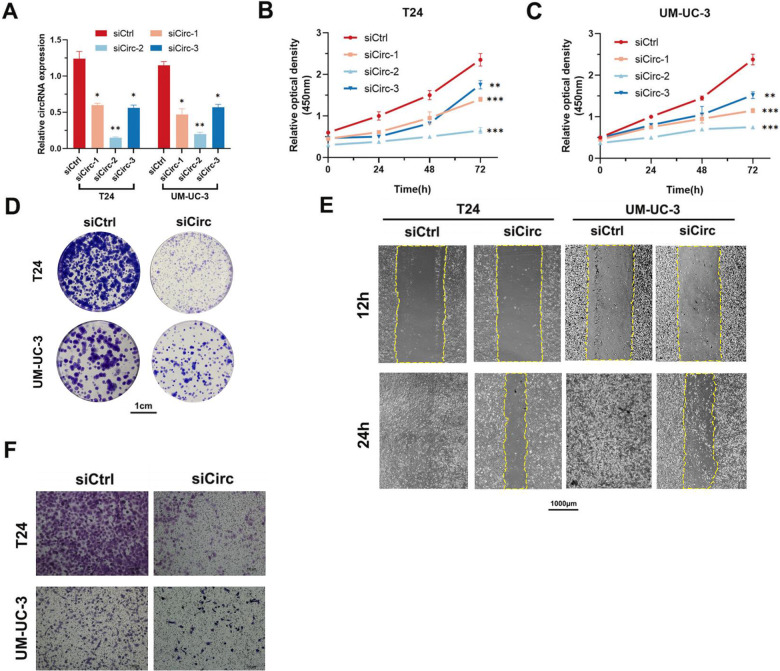

**Figure:**