# Supplementary material for: Autophagy-associated circular RNA hsa_circ_0007813 modulates human bladder cancer progression via hsa-miR-361-3p/IGF2R regulation
Source: Cell Death Dis. 2021 Aug 7;12(8):778. doi: 10.1038/s41419-021-04053-4 (PMC8349354; doi:10.1038/s41419-021-04053-4)
Supplement: Supplementary file 1 — Supplementary tables [file 41419_2021_4053_MOESM1_ESM.docx]

Supplementary tables

Supplementary table S1

Up-regulated circular RNAs ranked by Log2FC/Deviation

| Ranking | circRNA_ID | log2FC | p_value | Ca-1 | Ca-2 | Con-1 | Con-2 | Log2FC/Deviation |
| --- | --- | --- | --- | --- | --- | --- | --- | --- |
| 1 |  | 6.633243 | 0.012429 | 541.6039 | 1373.602 | 0 | 0 | 1.150967541 |
| 2 |  | 6.322366 | 0.023264 | 1191.529 | 343.4005 | 0 | 0 | 0.904892357 |
| 3 | hsa_circ_0007813 | 6.099897 | 0.037368 | 1083.208 | 228.9337 | 0 | 0 | 0.76798624 |
| 4 |  | 6.414972 | 0.01962 | 1408.17 | 228.9337 | 0 | 0 | 0.694137237 |
| 5 |  | 6.366288 | 0.022458 | 216.6415 | 1373.602 | 0 | 0 | 0.687250585 |
| 6 |  | 6.497536 | 0.016699 | 1733.132 | 0 | 0 | 0 | 0.5 |
| 7 |  | 5.995881 | 0.044502 | 649.9246 | 572.3342 | 0 | 0 |  |
| 8 |  | 5.715976 | 0.003578 | 4116.189 | 0 | 58.3285 | 0 |  |
| 9 |  | 5.07286 | 0.002477 | 4657.793 | 0 | 58.3285 | 61.46488 |  |
| 10 |  | 4.798535 | 0.02575 | 2166.415 | 0 | 58.3285 | 0 |  |
| 11 |  | 4.45668 | 0.001837 | 18522.85 | 343.4005 | 349.971 | 491.7191 |  |
| 12 |  | 3.876844 | 0.028379 | 108.3208 | 1945.936 | 0 | 122.9298 |  |

Supplementary table S2

Ranked downstream genes of hsa-miR-361-3p

| ensembl_gene_id | hgnc_symbol | gene_biotype | p.val | p.val_adjusted |
| --- | --- | --- | --- | --- |
| ENSG00000134531 | EMP1 | protein_coding | 0.000465904 | 0.016306634 |
| ENSG00000197081 | IGF2R | protein_coding | 0.005972823 | 0.104524405 |
| ENSG00000135472 | FAIM2 | protein_coding | 0.023198935 | 0.192693507 |
| ENSG00000181449 | SOX2 | protein_coding | 0.027527644 | 0.192693507 |
| ENSG00000159388 | BTG2 | protein_coding | 0.021640355 | 0.192693507 |
| ENSG00000129244 | ATP1B2 | protein_coding | 0.069919862 | 0.407865864 |
| ENSG00000132535 | DLG4 | protein_coding | 0.121526266 | 0.484980233 |
| ENSG00000169194 | IL13 | protein_coding | 0.124709203 | 0.484980233 |
| ENSG00000069702 | TGFBR3 | protein_coding | 0.108923578 | 0.484980233 |
| ENSG00000170027 | YWHAG | protein_coding | 0.157446747 | 0.551063614 |
| ENSG00000119689 | DLST | protein_coding | 0.191926227 | 0.610674358 |
| ENSG00000198752 | CDC42BPB | protein_coding | 0.221505029 | 0.646056335 |
| ENSG00000188263 | IL17REL | protein_coding | 0.250119069 | 0.673397492 |
| ENSG00000106244 | PDAP1 | protein_coding | 0.297360583 | 0.743401457 |
| ENSG00000180304 | OAZ2 | protein_coding | 0.366620293 | 0.855447351 |
| ENSG00000169992 | NLGN2 | protein_coding | 0.463201042 | 0.855544209 |
| ENSG00000198198 | SZT2 | protein_coding | 0.546892205 | 0.855544209 |
| ENSG00000109787 | KLF3 | protein_coding | 0.464895091 | 0.855544209 |
| ENSG00000101384 | JAG1 | protein_coding | 0.479555148 | 0.855544209 |
| ENSG00000158195 | WASF2 | protein_coding | 0.521178534 | 0.855544209 |
| ENSG00000068971 | PPP2R5B | protein_coding | 0.566160179 | 0.855544209 |
| ENSG00000172757 | CFL1 | protein_coding | 0.571975955 | 0.855544209 |
| ENSG00000174576 | NPAS4 | protein_coding | 0.582555629 | 0.855544209 |
| ENSG00000053108 | FSTL4 | protein_coding | 0.586658886 | 0.855544209 |
| ENSG00000099364 | FBXL19 | protein_coding | 0.63851248 | 0.878618555 |
| ENSG00000139645 | ANKRD52 | protein_coding | 0.655324348 | 0.878618555 |
| ENSG00000144579 | CTDSP1 | protein_coding | 0.677791457 | 0.878618555 |
| ENSG00000159217 | IGF2BP1 | protein_coding | 0.741738512 | 0.895201653 |
| ENSG00000163251 | FZD5 | protein_coding | 0.726935299 | 0.895201653 |
| ENSG00000126012 | KDM5C | protein_coding | 0.916374423 | 0.977817449 |
| ENSG00000171791 | BCL2 | protein_coding | 0.892915147 | 0.977817449 |
| ENSG00000148719 | DNAJB12 | protein_coding | 0.884921241 | 0.977817449 |
| ENSG00000105767 | CADM4 | protein_coding | 0.921942166 | 0.977817449 |
| ENSG00000170265 | ZNF282 | protein_coding | 0.98746226 | 0.994726004 |
| ENSG00000100314 | CABP7 | protein_coding | 0.994726004 | 0.994726004 |

Supplementary table S3

|  | Sequence (DNA/RNA) |
| --- | --- |
| hsa_circ_0007813 FISH probe | TTTTCGTCGACCGGACGTTCAACTAGTACCGGAACGGTCC |
| hsa_circ_0007813 RNA pull-down probe | UUGAUCAUGGCCUUGCCAGGUUGGUGACAGUAUAUUGUGAGCAUGGUCAUAAAGCUGCCAAAAUCAACCCCCUCUUCACCGGACAAGCCCUGCUGGAGAAUGUGCCUGAAAUCCAAGCCCUGGUGCAGACACUGCAGGGACCCUUCCACACGGCAGGAUUAUUGAACAUGGGGAAGGAAGAGGCCUCACUUGAGGAAGUGUUAGUCUAUCUCAAUCAAAUCUACUGUGGGCAGAUUUCUAUUGAAACCUCCCAACUUCAGAGCCAGGAUGAGAAAGACUGGUUUGCCAAGCGGUUUGAGGAACUGCAAAAGGAGACGUUUACCACAGAAGAGCGAAAACAUCUGUCGAAACUAAUGCUGGAAUCUCAGGAGUUUGACCACUUUCUGGCCACCAAGUUCUCGACAGUGAAGCGAUAUGGAGGCGAAGGGGCUGAAAGCAUGAUGGGCUUUUUCCACGAGCUGCUGAAAAUGUCGGCCUACAGCGGGAUCACUGAUGUCAUUAUUGGGAUGCCCCAUAGAGGGAGGCUGAAUUUAUUGACAGGCCUUCUGCAGUUCCCUCCAGAGCUGAUGUUCCGUAAAAUGCGAGGCUUAAGUGAAUUUCCAGAGAAUUUCUCAGCCACUGGAGACGUCCUGUCUCACCUGACCUCCUCUGUGGACCUGUACUUUGGGGCGCACCAUCCCCUCCAUGUGACAAUGUUGCCCAAUCCCUCGCACCUGGAGGCCGUCAACCCCGUGGCCGUGGGCAAAACUCGCGGCAGGCAGCAGUCUCGCCAAGACGGCGAUUACUCUCCAGACAACUCAGCCCAGCCGGGGGACAGGGUCAUUUGCUUACAGGUCCAUGGUGAUGCUUCUUUCUGUGGUCAAGGGAUUGUUCCUGAAACAUUCACGCUGUCCAAUCUCCCACAUUUCAGAAUUGGUGGGAGUGUGCAUUUGAUUGUUAAUAACCAGCUGGGUUACACCACUCCAGCUGAAAGAGGAAGGUCUUCUUUAUACUGCAGUGAUAUUGGGAAGCUUGUGGGCUGUGCCAUCAUCCAUGUCAAUGGAGACAGCCCAGAGGAAGUGGUCCGUGCCACACGACUGGCUUUUGAAUACCAACGCCAGUUCCGCAAGGAUGUGAUUAUUGAUCUGUUGUGCUACAGGCAGUGGGGCCACAAUGAGCUGGAUGAGCCAUUCUACACCAACCCCAUCAUGUACAAAAUCAUCAGAGCUCGAAAGAGCAUUCCAGACACAUAUGCAGAGCACCUCAUUGCUGGCGGACUCAUGACGCAGGAGGAGGUGUC  UGAAAUAAAAUCCUCCUACUAUGCCAAGUUGAAUGAUCACUUAAAUAACAUGGCCCACUACAGGCCCCCUGCCCUGAACCUGCAGGCCCACUGGCAGGGCCUGGCUCAGCCAGAAGCGCAAAUCACCACCUGGAGUACAGGUGUGCCCCUCGACCUCCUGCGGUUUGUUGGCAUGAAGUCUGUAGAGGUGCCAAGAGAGCUGCAGAUGCACAGUCACCUGCUGAAGACACAUGUUCAGUCCAGAAUGGAGAAGAUGAUGGACGGAAUCAAGCUAGACUGGGCCACCGCGGAAGCUCUUGCCUUGGGUUCUUUACUUGCUCAAGGUUUUAAUGUUCGUCUAAGUGGCCAAGAUGUUGGUCGUGGAACUUUCAGUCAGAGGCAUGCAAUCGUGGUUUGCCAGGAGACGGAUGACACCUACAUCCCCCUGAACCAUAUGGACCCAAAUCAGAAGGGGUUUCUAGAGGUCAGCAACAGCCCACUGUCAGAAGAGGCCGUCCUGGGAUUUGAAUAUGGGAUGAGCAUUGAGAGCCCAAAGUUACUGCCCCUGUGGGAGGCACAGUUUGGCGAUUUCUUCAAUGGUGCCCAGAUCAUCUUUGACACAUUCAUCUCUGGAGGAGAGGCCAAGUGGCUCCUACAAAGCGGCAUCGUCAUCCUCCUUCCACAUGGCUACGAUGGGGCUGGGCCAGACCACUCAUCCUGUCGAAUAGAGCGUUUCCUGCAGAUGUGUGACAGUGCGGAAGAGGGGGUGGACGGAGACACUGUGAACAUGUUUGUGGUUCACCCAACAACUCCUGCACAGUAUUUCCACUUGCUUAGGAGACAGAUGGUCCGGAACUUCAGAAAACCACUCAUUGUUGCUUCCCCUAAGAUGUUACUCAGGCUCCCGGCAGCCGUGUCAACUCUUCAAGAAAUGGCACCAGGAACAACAUUUAACCCGGUCAUUGGUGAUUCAUCUGUGGAUCCAAAAAAGGUUAAGACCCUCGUGUUCUGCUCCGGCAAACAUUUCUACUCCCUGGUGAAACAAAGAGAAUCUCUGGGGGCCAAGAAGCAUGACUUUGCCAUCAUCCGAGUAGAGGAACUCUGCCCCUUCCCGUUGGAUUCUUUACAGCAAGAGAUGAGCAAAUACAAACAUGUUAAAGAUCAUAUUUGGAGUCAGGAGGAACCUCAGAACAUGGGUCCGUGGUCGUUUGUUUCUCCAAGGUUUGAAAAGCAGCUGGCCUGCAAG |
| hsa-miR-361-3p inhibitor | AAAUCAGAAUCACACCUGGGGGA |
| hsa-miR-361-3p mimic | UCCCCCAGGUGUGAUUCUGAUUU  AUCAGAAUCACACCUGGGGGAUU |
| miRNA inhibitor control | CAGUACUUUUGUGUAGUACAA |
| hsa_circ_0007813 siRNA | AGCUGGCCUGCAAGUUGAUTT  AUCAACUUGCAGGCCAGCUTT |
| siRNA control | UUCUCCGAACGUGUCACGUTT  ACGUGACACGUUCGGAGAATT |
| Mimic control | UUCUCCGAACGUGUCACGUTT  ACGUGACACGUUCGGAGAATT |
| IGF2R F primer | GTTGCCCTCCAGAAACCGAT |
| IGF2R R primer | CGTAGTCCGCAGTTGTGCTA |
| hsa_circ_0007813 F primer | CAGCTGGCCTGCAAGTTGA |
| hsa_circ_0007813 R primer | GCACCAGGGCTTGGATTTC |
| GAPDH F primer | CATGAGAAGTATGACAACAGCCT |
| GAPDH R primer | AGTCCTTCCACGATACCAAAGT |
