# Supplementary figures and images for: Autophagy-associated circular RNA hsa_circ_0007813 modulates human bladder cancer progression via hsa-miR-361-3p/IGF2R regulation
Source: Cell Death Dis. 2021 Aug 7;12(8):778. doi: 10.1038/s41419-021-04053-4 (PMC8349354; doi:10.1038/s41419-021-04053-4)

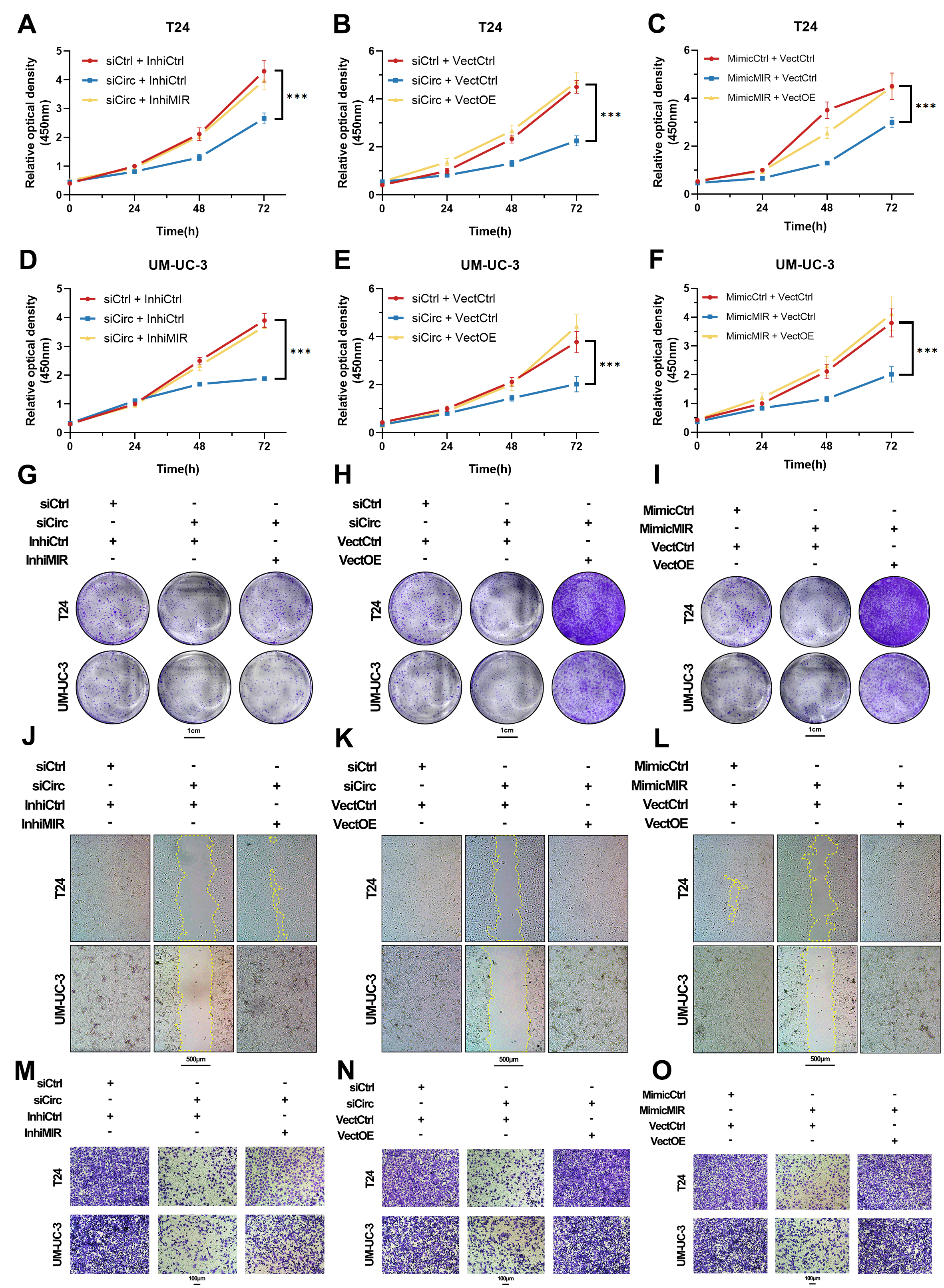

Supplement: Supplementary file 2 — Supplementary figure S1 [file 41419_2021_4053_MOESM2_ESM.png]
