# Supplementary material for: Autophagy-associated circular RNA hsa_circ_0007813 modulates human bladder cancer progression via hsa-miR-361-3p/IGF2R regulation
Source: Cell Death Dis. 2021 Aug 7;12(8):778. doi: 10.1038/s41419-021-04053-4 (PMC8349354; doi:10.1038/s41419-021-04053-4)
Supplement: Supplementary file 3 — Legend of supplementary figure S1 [file 41419_2021_4053_MOESM3_ESM.docx]

**Supplementary figure legend**

**Supplementary figure S1**

**CircRNA hsa_circ_0007813 acted as a sponge for hsa-miR-361-3p, a micro-RNA targeting IGF2R.**

After silencing hsa_circ_0007813 with siRNA transfection (siCirc), T24 and UM-UC-3 cells were further transfected with hsa-miR-361-3p miRNA inhibitor (InhiMIR). These cells were subjected to (A and D) CCK-8 assays (N = 3, Kruskal-Wallis test), (G) colony formation assays, (J) wound healing assays and (M) Transwell assays. The siRNA-transfected cells (siCirc) were further transfected with IGF2R overexpression vectors (VectOE). These cells were subjected to (B and E) CCK-8 assays (N = 3, Kruskal-Wallis test), (H) colony formation assays, (K) wound healing assays and (N) Transwell assays. After hsa-miR-361-3p miRNA mimic (MimicMIR) transfection, cells were further transfected with IGF2R overexpression vectors (VectOE). These cells were subjected to (C and F) CCK-8 assays (N = 3, Kruskal-Wallis test), (I) colony formation assays, (L) wound healing assays and (O) Transwell assays. All error bars in figures indicated the standard deviation of three independent experiments. * P < 0.05, ** P < 0.01, *** P < 0.001. Scale bars in G, H and I represent 1cm. Scale bars in J, K and L represent 500μm. Scale bars in M, N and O represent 100μm.
